# Living through the COVID-19 pandemic in Mauritius: mental well-being and dependence on Facebook

**DOI:** 10.1007/s44202-022-00044-4

**Published:** 2022-07-01

**Authors:** Shilpa Ramdawor, Manish Putteeraj, Numrata Moty, Jhoti Somanah

**Affiliations:** School of Health Sciences (SHS), University of Technology, Mauritius, Pointe aux Sables, La Tour Koenig, Mauritius

**Keywords:** COVID-19, Youth, Facebook usage, Addiction, Anxiety, Isolation, Mental well-being

## Abstract

**Background:**

The unprecedented outbreak of the Coronavirus disease (COVID-19) resulted in numerous psychological consequences among young Mauritians. Prominently, an increase in Facebook usage during the pandemic was observed which could influenced the mental well-being of Facebook users.

**Objective:**

The current study sought to explore how the pattern of use, as well as the purpose of Facebook usage, could impact the mental well-being of young Mauritian adults, before, during and after the pandemic-mediated confinement.

**Method:**

A cross-sectional approach using a sample of 378 young adults was chosen with a self-administered questionnaire shared through online mediums. The instrument consisted of a combination of validated scales and self-developed items.

**Results:**

The findings revealed a radical proliferation of social media (91%) through a self-perceived dependency for its informative purpose and related addiction; as well as an evolution of adverse psychological effects characterized by a spectrum of feelings such as restlessness and lowered self-esteem. Higher scores of depressive symptoms were observed during the confinement period (10.05 ± 0.13) as opposed to pre- (0.31 ± 0.79) and post- (0.38 ± 0.09) temporal zones. The lowest scores of mental well-being were noted during the confinement period (0.77 ± 1.8) as compared to pre-confinement (6.56 ± 1.42) and a remarkable recovery was observed post the confinement phase (6.68 ± 1.32). Changes in emotional states were also identified as important predictors of Facebook addiction (χ^2^(1) = 94.54, *p* < 0.001) with 48.8% of the variation in the reported addiction behavior matched with 92.6% of perceived addictive characteristics.

**Conclusion:**

Facebook dependency during the lockdown period was paired with a number of adverse psychological effects among young Mauritians; effects which were likely associated with the frequency and purpose of Facebook use during the pandemic.

**Supplementary Information:**

The online version contains supplementary material available at 10.1007/s44202-022-00044-4.

## Introduction

Significant advancements in networking strategies have emerged with evolving technology rendering communication more accessible, triggering the uprise of virtual social networking sites. The fundamental aspect of social media lies in its role as a mediator, enabling users to mutually relate through virtual social networking and catalyzing the establishment of virtual communities, primarily tasked with the exchange of views, opinions, information, knowledge, and simplifying professional networking [59]. In 2004, the most prevalent social networking site, currently known as ‘Facebook’ was created which now forms part of a bigger brand ‘Meta’. Facebook has a global member profile estimated at 2.9 billion which by far justifies the research focus on this particular platform [26]. Moreover, even in the local context, Facebook has been statistically reported as the most preferred social networking platform with 87.3% dominance over other networking sites [77]. Over the years, the adaptive features of Facebook have captured subscribers as demonstrated by the mesh-like design encouraging its users to maintain prevalent relationships through group networking while allowing some degree of accountability to deter deceptive endeavors [17].

An overdependence on technology and the virtualization of social interactions has led to the erosion of social skills such as empathy [81]. Heavy Facebook usage has been associated with personal and professional neglect and impaired social responsibilities [87]. On average, individuals, mostly females within the younger age cluster spend 75.2 min daily, accounting for 84% of their daily routine on Facebook [21]. The increased dependency on virtual social and emotional support, although subjective across gender and age groups, can be detrimental especially for ‘influencers’ who are often perceived as ‘opinion leaders’ or role models [20, 39]. Dimensions such as vitality, life satisfaction and happiness are significantly influenced by problematic Facebook use- further advocating the plural role of Facebook misuse on the subjective individual [66].

The impact of social media and social networking sites’ dependence on the psychological status and mental well-being at large should not be downplayed. Kuss and Griffiths [41] revealed that addiction to Facebook often leads to experiencing symptoms similar to those suffering from addictive disorders. Changes in social media and social networking sites’ usage patterns and purposes can precipitate a number of mental well-being issues namely, symptoms of major depressive disorders, compulsive disorders, bipolar disorders, dysthymia, narcissistic and antisocial disorders, and addiction among others; conditions which have been regrouped and coined as ‘IDisorder’ [61, 62]. Xu and Tan [86] reported that the transition from normal social networking use to misuse arises when social networking is believed to be a significant, if not exclusive instrument to cope with loneliness, stress, and depression. This has been supported by the findings of Dibb and Foster [24], acknowledging the association between social isolation and the type of Facebook usage. Intense Facebook use has been identified as a predictor of loneliness [42], and its impact on psychosocial functioning vary from decreased sleep quality and impeded overall health to relatively higher stress [3, 85]. Further mapping of those disorders against the age spectrum has shown that youths with a higher engagement rate with respect to addressing and checking social media alerts (continuously every fifteen minutes) tend to score higher on the anxiety scale as compared to the elderly [62]. Facebook addiction is also linked to the inability to provide emotional support and manage interpersonal conflicts in adolescents, potentially leading to peer alienation and emotional dysregulation, decreased well-being as well as increased psychiatric distress [55]. Hence, early identification of behavioral issues, which are tied to social networking dependence and misuse, may be effective in mitigating the long-lasting effects inflicted on the individual.

Even though Facebook addiction was a behavioral concern prior to the coronavirus disease (COVID-19) pandemic, a significant media dependency has been noted post the outbreak [48]. The sanitary measures implemented to contain the spread of the disease in the form of quarantine, lockdowns and restricted movements led to an increase in internet usage. This consequently increased the risk of problematic internet use the latter characterized as poorly controlled use of the internet in the form of excessive screen time, addiction to social media, watching pornography, online gambling, and video gaming as stress or anxiety relief measure [23, 37]. Paradoxically, while isolation reinforced feelings of loneliness and increased the need for virtual communities to bridge the COVID-19 induced-socialization gap, the virtualization dependence compounded negative consequences [9] as further demonstrated through feelings of rash impulsiveness over the reward drive being associated to Facebook addiction [30].

A bi-directional relationship has also been identified through quantitative research strategies whereby sedentary endeavors causing anxiety, depression, loneliness, stigma, and violence among others, emerged from the pandemic-mediated confinement as reinforcement factors toward Facebook addiction [28, 30]. Interestingly, self-monitoring and regulation of screen time have been considered essential since constant verification of social media and watching news updates about the pandemic negatively influenced mental well-being [5]. Statistically larger psychological flexibility and acceptance of difficult experiences appear to act as buffers against the negative effects of increased social isolation and concomitantly intensify the advantages of social connectivity, endorsing the role of precursor mental well-being traits as a protective factor against isolation-induced consequential effects [76].

Globally, the effects of Facebook usage in terms of its exposure, use pattern, as well as social networking styles driving mental health conditions are well characterized. However, such data in the Mauritian context is scant especially when considering the extent to which social isolation caused by the recent confinement might have triggered an overdependence on Facebook and its psychological repercussions for its majority of users. Even though statistical information such as age and gender disparities of Facebook users are recorded, there is hardly any contemporary research aimed at understanding mental well-being in relation to their Facebook usage. Mauritius, being an island destination with a proliferating westernized culture, has not witnessed such global health disasters leading to country-wide confinement and social isolation since the advent of social networking sites and social media. Hence, the subjective influence of the unprecedented pandemic on young Mauritian Facebook users, along with its consequential impact on mental well-being would provide vital information on the behavioral variations, if any, of individuals originating from island destinations where socialization is at the heart of their undertakings. This present study aims to determine the influence of the pandemic on Facebook usage and mental well-being through a cross-sectional study based on pre, during, and post-pandemic sentiments; supported through a quantitative approach and probabilistic sampling strategies.

## Methods

### Participants

The sample for this study consisted of individuals aged between 18 and 32 years old with a personal Facebook account, used on a regular basis at least once weekly. Additionally, care was taken to ensure that no professional Facebook accounts were chosen during data collection. Facebook was chosen because of its highest usage prevalence, more precisely 91.5% [77] as compared to other social networking sites. Given that the majority of Mauritian Facebook users were estimated to belong to the age group of 18–32 years (54.1%) as of January 2020; only those aged 18 to 24 (190,840; 22.4% of 852,000) and those between 25 to 32 years, (270,084; 31.7% of 852,000), were considered the target population thus narrowing it down from 852,000 to 460,924 [77]. Furthermore, a sample size of 400 was calculated based on Slovin’s formula, taking into account a margin error of 5% and a confidence interval of 95%. A final response rate of 94.5% was observed with 378 valid participants.

Each participant was briefed prior to the start of the survey with respect to the confidential management of the collected data. The survey was accompanied by a cover page whereby participation consent was sought. Information was kept anonymous at all times and the ethical standards were respected. Ethical requirements were vetted by the Postgraduate Dissertation Committee, School of Health Sciences, University of Technology, Mauritius.

### Materials

A questionnaire was designed based on the core variables of the study, i.e., mental well-being, Facebook usage and pattern; and social media addiction scale. Items for the sections were either presented in a dichotomous, polychotomous, Likert, or rating profile to capture a wider range of behavioral attributes and perceptions from the study participants. Scales that were consulted were either used verbatim or adapted for the purpose of the study. Given that this area of research is still fairly new in the Mauritian context, no scales related to study variables have been validated for the local landscape. However, the scales were carefully chosen based on similarities in culture and research scope. The Bergen Facebook Addiction Scale (BFAS) which specifically addresses addiction to the social networking site has been adapted to the Indian context [11, 46]. Likewise, the Short Warwick Edinburg Mental Well-Being Scale (SWEMWBS) has been prominently used and translated in several Indian studies both scales satisfy the requirements in the local context given the high proportion of Indo-Mauritians approximating 66% and the similarities between the two cultures [71, 83]. Socio-demographic data of the participants were examined in the first section of the questionnaire with the addition of items such as educational level and income.

#### Psychological status

This section consisted of three scales. Firstly, an adaptation of the ‘The Assessing Emotions Scale’ [70] to used to determine the current emotional state of participants, using a 10-point Likert scale (1 = depressed to 10 = dynamic). Secondly, a modified version of the Short Warwick Edinburg Mental Well-Being Scale (SWEMWBS) [78] was used to evaluate mental-well being of participants given its sensitivity towards meaningful clinical changes based on a 4-point Likert scale (1 = Strongly Disagree to Strongly Agree). This section included questions that were converted into a dichotomous profile to facilitate response and included questions to reinforce the structure for mental well-being assessment.

#### Social networking sites usage and pattern

This section was designed to measure social networking site preferences, internet access, exposure time and activities engaged on social networking sites. The multiple-choice questions were developed and adapted for the purpose of this study based on the salient features of the population with respect to the most common platforms used in the Mauritian context. This section also included generic items formulated based on usage frequency and patterns from previous literature.

#### Psychological impact based on social networking sites’ use

A 2-tiered assessment was favored whereby the initial portion was a convergence of extracts from the Social media Addiction Scale Student Form (SMAS-SF) [64], Bergen Facebook Addiction Scale (BFAS) [2] and the Brief Symptom Inventory Dimensions Scales [8, 22] measured on a 4-point Likert profile (1 = Very Rarely to 4 = Very Frequently) to map salience, mood modification, tolerance, withdrawal, conflict, and relapse; as well as lifestyle behavioral modifications. The other segment relied on dichotomous items, more precisely ‘Yes’ or ‘No’ responses categorized into pre-, during- and post-confinement phases.

### Procedures

A quantitative study using a probabilistic approach was conducted over 6 months (September 2020 to March 2021) through virtual mode given the prevailing sanitary measures in the country. SPSS V22.0 for Windows was used for data entry and processing. Nominal data were expressed as percentages and frequency. Statistical significance was assessed at *p* < 0.05. Variation in data distribution was evaluated using the Shapiro–Wilk test with a cut-off value of *p* > 0.05, designating the normality of data. Further inferential analysis was undertaken to identify plausible relationships using the Chi-Square test and directionality of association through correlational analysis; both with reported measures of strength. Comparative tests were also undertaken using the Mann–Whitney U tests which were also coupled with the Kruskal Wallis H test to determine the effect of the independent variable of interest such as the temporal variants on mental wellbeing.

## Results

### Respondents’ profile and Facebook usage pattern

The National Statistics’ report on local Facebook users for March 2021 revealed that males were 52%, while females were 48% of the entire population of Facebook users in the country [51] data which mostly tallied with our present sampled population with a margin of 4% across gender, which is almost thrice as much in the actual population, whereby the gender difference is 5.8%. Likewise, with respect to the age strata, the findings are more or less comparable to the population of Facebook users’ statistics, with the subgroup of ‘25–34’ being the largest user group with 30.9% 77] in comparison to the 23–27 age cluster in our study with 24.5% and the 23–32 age category accounting for a total of 75%. Most respondents (n = 374) were educated above the primary level, while only 24% were either unemployed or students. Those in a professional stream and earning twice the national minimum wage accounted for 67% of the sample (Table 1).

**Table 1:** Participants’ characteristics

Description	Frequency (n =)	Percentage (%)
Gender		
Male	185	46.25
Female	193	48.25
Age		
18–22	100	25
23–27	98	24.5
28–32	180	50.5
Relationship status		
Single	87	21.75
In a relationship	105	26.25
Married	102	25.5
Divorced	62	15.5
Widowed	22	5.5
Highest education		
Primary	2	0.5
Secondary	34	8.5
Tertiary	201	50.25
Professional Qualifications	141	35.25
Occupation		
Student	72	18
Self-employed	107	26.75
Employed	174	43.5
Unemployed	20	5
Income range		
Rs 10,000–Rs 20,000	33	8.3
Rs 20,001–Rs 30,000	127	31.8
Rs 30,001–Rs 40,000	99	24.8
Rs 40,001–Rs 50,000	24	6.0
Rs 50,001+	4	1.0
None	91	22.8

**Table 2:** Exposure to social media and Facebook usage patterns

Items	Frequency (n =)	Percentage (%)
Preferred Social Media platform		
Facebook	344	91.0
Instagram	4	1.0
Others	30	8.0
Total estimated time spent on Facebook (Daily)		
< 1 h	0	0
1–2 h	19	5.0
2–3 h	49	13.0
3–6 h	132	35.0
> 6 h	178	47.0
Facebook usage pattern across the day		
Morning to Noon (6 am to 12 pm)	2	0.5
Afternoon only (12 pm to 6 pm)	24	6.4
Evening (6 pm to 12 am)	119	31.6
Throughout the day (6 am to12 am)	233	61.5
What are the main activities which can be undertaken through Facebook?		
Educational purposes	0	0
Information (News etc..)	45	12.0
Social interaction (connectivity)	329	87.0
Personal expression	4	1.0
Lifestyle inspiration	0	0

The present data showed that the majority of respondents (91%) had an active subscription to Facebook, with a lower percentage also using other platforms such as TikTok to access social media content. This tallied with the accessibility to internet connectivity through various devices, mostly mobile phones (95.8%) tailed by computer/laptop devices (3.7%). Considering the inclusion criteria of internet access, most of the respondents affirmed that they had unlimited internet access explaining the ability of the respondents to log onto the platform throughout the day (61.5%) mostly for connecting with peers (n = 322) and to a lesser extent creating contents (n = 30) or browsing (n = 13). A certain category would mostly prefer signing onto Facebook in the evening period from 6 pm to 12 am which to a certain extent would potentially overlap with their marital status or personal and professional commitments during the daytime (Table 2).

### Respondents’ perceived addiction to Facebook

Reviewing their activity on Facebook, a major proportion of the respondents defined themselves as addicted to the social media platform (91%, n = 343) with 90.7% reporting a self-rated connectedness profile of above 6 on a scale of 0–10 (Ẋ = 8.06 ± 1.11); perceived addiction which was not gendered (*p* > 0.05). This was also in agreement with their need to ‘check’ their Facebook page as a source of motivation (97.1%, n = 367) to get by with their daily activities. Using selected items from the Social Media Addiction Scale and the Bergen Facebook Addiction Scale (Table 3; α = 0.874) it could be observed that the majority of respondents (86.5%, n = 327) felt anxious if they did not respond to their Facebook notifications which also tallied with their general inability to reduce their Facebook usage (86.8%, n = 328). This was further reinforced by the positive relationship identified between (i) perceived addiction versus Facebook usage (χ^2^(3) = 129.13, *p* < 0.001; Cramer’s V = 0.585) and (ii) daily total estimated time spent versus Facebook usage (χ^2^(12) = 214.48, *p* < 0.001; Cramer’s V = 0.435); with a reported 177 respondents spending more than 6 h daily and finding it difficult to reduce their usage.

**Table 3:** Assessing behavioral cues of addiction with respect to Facebook during the COVID-19 period

Description	Very rarely	Rarely	Often	Very frequently
Ignoring Facebook notifications leads to anxiety	2.8	5.3	52.5	34.0
Facebook stimulates my intellectual and/or emotional traits	1.0	4.8	53.0	35.8
I cannot reduce my Facebook usage	1.8	6.0	52.3	34.5
I catch up on news and information on Facebook	0	2.8	50.0	41.8
I have interpersonal conflicts due to my dedication to Facebook	19.3	32.8	28.3	14.3
Facebook causes fulfillment through creative self-expression	1.5	4.5	53.0	35.5

*Data reported as percentage (%) of total number of respondents

### Living through the COVID-19 pandemic: impact on mental wellbeing

Using a modified version of the Short Warwick Edinburg Mental Well-being Scale (SWEMWBS), the respondents’ self-reported assessment of their mental well-being was recorded for the temporal period spanning the first COVID-19-mediated confinement from March to May 2020 (Table 4). Given that data collection was initiated in September 2020, respondents had a recovery period of 3 months from the confinement effects. Interestingly, when taking into consideration the weighted scores across the 3 blocks, i.e., pre, during, and post confinement, the present data demonstrated a strong impact of the sudden confinement measures on mental wellbeing (pre: 6.56 ± 1.42; during: 0.77 ± 1.8; and Post: 6.68 ± 1.32; *scores closer to 7 meant inferred positive mental wellbeing*). A significant effect of the COVID-19-mediated confinement was also identified on mental wellbeing through the Kruskal Wallis H test (χ^2^(2) = 843.27, *p* < 0.001); with a drastic decrease during the confinement period (During vs. Pre; Z = − 16.94, *p* < 0.001) and recovery post lifting of measures (During vs. Post: Z = − 16.6, *p* < 0.001).

**Table 4:** Mental wellbeing status through the first COVID-19 mediated confinement

Description	Pre confinement			During confinement		Post confinement	
		Freq	%	Freq	%	Freq	%
Optimistic about the future	Yes	357	89.3	25	6.3	339	84.8
	No	21	5.3	339	84.8	22	5.5
Feeling useful	Yes	359	89.8	32	8.0	348	87.0
	No	17	4.3	333	83.3	14	3.5
Feeling relaxed	Yes	349	87.3	46	11.5	338	84.5
	No	29	7.3	319	79.8	21	5.3
Been dealing with problems well	Yes	355	88.8	38	9.5	343	85.8
	No	23	5.8	326	81.5	15	3.8
Been thinking clearly	Yes	352	88.0	39	9.8	344	86.0
	No	26	6.5	323	80.8	15	3.8
Been feeling close to others	Yes	348	87.0	57	14.3	340	85.0
	No	30	7.5	305	76.3	19	4.8
Been able to make up my own mind	Yes	353	88.3	53	13.3	342	85.5
	No	25	6.3	308	77.0	17	4.3

^*^Data presented in Frequency (n =) and percentage (%) based on the total number of respondents for the individual items

**Fig. 1:**
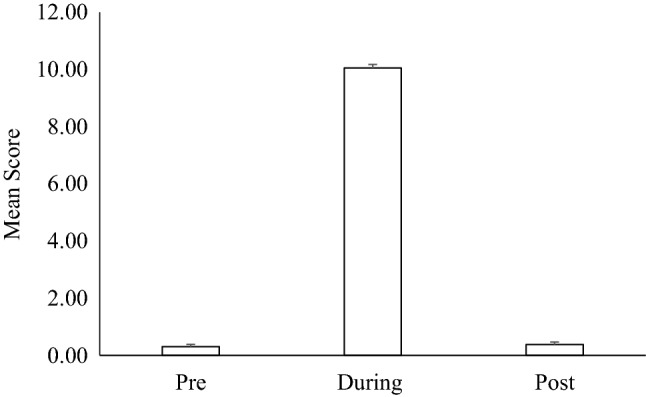
Mean score recorded for the Short Mood and Feeling Questionnaire. The mean score recorded were as follows; Pre: 0.31 ± 0.79; during: 10.05 ± 0.13; and Post 0.38 ± 0.09. Data presented as mean ± SEM. (The 11-item questions were scored with a maximum score of 13 and a minimum of 0; higher the score would indicate greater severity of depressive symptoms with a cut-off value of 7)

Mental well-being was further assessed using the Short Mood and Feelings Questionnaire (SMFQ) over the temporal period covering the onset of the sanitary measures mediated by the COVID-19 outbreak locally (Supplementary S1). A significant confinement-driven effect on mood and feelings was noted ((χ^2^(2) = 917.59, *p* < 0.001). It was also observed that the sudden change in mobility and barriers to socialization instilled by the confinement led to a drastic increase in the SMFQ scores recorded for the participants as opposed to the period before and after the movement control ((During vs. Pre: Z = − 17.33, *p* < 0.001; During vs. Post: Z = − 17.30, *p* < 0.001; and Pre vs. Post: Z = − 1.60, *p* = 0.11); statement which was supportive of the high percentage response (> 80%) to items such as ‘*miserable and unhappy’* or ‘*feeling lonely’* during confinement, contrasting to the low association with such feelings prior and post confinement (< 5%). The present data hence reflects an increase in depressive symptoms initiated due to the confinement measures which were normalized after the lifting of those restrictions (Fig. 1).

### Facebook and mental well-being during the COVID-19 mediated confinement

A significant relationship was identified between self-reported Facebook addiction and emotional state (χ^2^(8) = 134.41, *p* < 0.001; Cramer’s V = 0.597); with emotions such as anger (n = 110), unworthiness (n = 92) and numbness (n = 67) being the most prevalent and accentuated during the COVID-19 mediated confinement. While social media and mental health share a bi-directional relationship; a binary logistic regression showed the ability to predict addiction to Facebook across a spectrum of emotional states ranging from feelings of depression to dynamism (χ^2^(1) = 94.54, *p* < 0.001) with the model generated explaining 48.8% of variation in the reported addiction behavior matched with 92.6% of perceived addictive characteristics. Furthermore, cross-tabulating feelings such as restlessness (Pre: χ^2^(3) = 9.88, *p* < 0.05; Cramer’s V = 0.162, During: χ^2^(3) = 82.18, *p* < 0.001; Cramer’s V = 0.480 and Post: χ^2^(3) = 4.03, *p* > 0.05); Cramer’s V = 0.597) and demotivation (Pre: χ^2^(3) = 11.86, *p* < 0.01; Cramer’s V = 0.177:, During: χ^2^(3) = 73.71, *p* < 0.001; Cramer’s V = 0.453 and Post: χ^2^(3) = 9.25, *p* < 0.05; Cramer’s V = 0.161) over the temporal conditioning of the COVID-19 mediated confinement against the inability to reduce time spent on Facebook showed that participants were already inclined to augmented usage of the social media platform prior to the confinement, but the association was revealed to be stronger during the confinement period.

## Discussion

The study aimed at understanding the relationship between Facebook use and mental well-being mediated by the COVID-19 pandemic. Based on previous reports, young adults aged between 18 to 32 years old were more prone to developing Facebook dependence; substantiating the target audience in this quantitative study which was conducted over 6 months with a sample size of 378 Facebook users. Interestingly, significant dependence on Facebook was noted during the lockdown period. With recent studies showing that Facebook usage can enhance its users’ well-being and socialization [12], the present findings validated this link as respondents perceived their dependence on Facebook as a source of motivation and part of their routine. However, this dependence was also associated with feelings of anxiety and restlessness which was more prominent during the social isolation period.

### Demographic variables and Facebook usage

 While Koc and Gulyagci [38] refute the idea of demographic factors acting as ‘*significant predictors’* of Facebook addiction; this notion was contrasted in the present study with consequential pointers on how demographic factors, namely age, educational level, and occupation can predict the usage of Facebook. Corroborating Ozimek and Bierhoff [53]’s findings, a linear relationship was observed between age and Facebook use, with the latter being more prominent among adults within the age group 28 to 32 years old. Villiers [84] reported the use of Facebook for its networking features as well as academic ventures through Facebook groups; tallying with the educational level of most respondents. A significant Facebook usage was also common among employed respondents which according to Robertson and Kee [58] is linked to a higher job satisfaction mediated by virtual social integration and professional connection.

### Facebook usage pattern and respondents’ perceived addiction

Corroborating Harpin et al. [33], the majority of respondents accessed their Facebook accounts through their smartphones. This increased use of smartphones over laptops is attributed to the cheaper cost of the former along with the modernization of mobile networks with low-priced cellular data packages, and ease of access [40, 56]. In line with the objective of this study, this change in technology use with an enhanced mobility option is more supportive of the prolonged usage of Facebook during the confinement period, a feature which would potentially be hindered in the absence of portable compact devices. Interestingly, while previous studies have reported the use of social media to be most prominent at night reflecting in-bed use of social networking sites [6], contradictory results were observed in this study with a significant use of Facebook throughout the day. Böhmer et al. [7] postulate the dominant use of social networking sites throughout the day as a result of the plurality in terms of features, i.e., ranging from content browsing to communication. Additionally, the present findings potentially align with the theory of “time of the day specific” content posited by Scherr and Wang [69], whereby specific time frames are allocated to the type of content being browsed or posted. This would account for trending information posted during the day, hence following a canonical pattern, while more novel information posted at night when individual attention is heightened towards the shared content. Coupled with the purpose of social media use, the habits which have emerged since the confinement period might not have changed after the movement restriction measures were lifted off, features which have been observed among Spanish adults [60].

The present findings of increased social media usage with a higher proportion attributed to Facebook are reflective of a global phenomenon induced by the pandemic [73]. Sanitary protocols such as compulsory use of face masks and social distancing during the COVID-19 pandemic led to major communication barriers, deterring willingness to engage in conversation without potentially increasing anxiety and stress features across the population [68]. The restrictive measures to contain the spread of the pandemic would have inevitably catalyzed the use of virtual communication, through social media and social networking sites as depicted by Saud et al. [67]. While healthy use of Facebook is deemed to be unproblematic for most users [36]; an inability to reduce Facebook usage as exemplified by over 6 h of dedicated ‘connectivity’ coupled with mood alterations when unable to respond to Facebook notifications as denoted in the present study points toward significant cues of onset or probable existing Facebook addiction [16, 63]. A high level of self-reported Facebook addiction with a negligible gender variation was also identified; findings which were further supported by Koc and Gulyagci [38] whereby interactions of gender on Facebook addiction are not significant predictors of Facebook addiction as opposed to other variables such as weekly commitments, social motives, and mental health status.

### Social media, Facebook, and mental well-being during the COVID-19 mediated confinement

The current findings were indicative of deteriorating mental well-being and increased depressive symptoms during the confinement period. The Mauritian population has never witnessed such a pandemic since the Spanish influenza in 1919, hence confirming the first-time experience of a pandemic for the targeted age group [57]. A sudden, unforeseen change in socialization habits with drastic confinement measures would presumably have a significant impact on mental well-being as implemented during the COVID-19-mediated lockdown in Mauritius. Changes of this magnitude resulted in significant feelings of loneliness which in turn potentially increased the risk of problematic internet use as reported by Deutrom et al. [23]. This poorly controlled use of the internet in the form of excessive screen time and addiction to social media [29, 54]; plausibly acted as coping strategies against anxiety and depressive moods during the confinement [37]. However, reliance on social media often loops into a vicious cycle with coping strategies exhibiting temporary ‘hormesis-like’ effects until uncontrolled usage aggravates feelings of loneliness and anxiety through virtual socialization and connectivity; in addition to worsening symptoms of existing disorders [4, 9, 14, 35, 72].

The notion of increased time spent on Facebook as reported in the present study could result from fear of COVID-19 as observed by Mannino et al. [44]. Dependency on Facebook during the pandemic was predominantly associated to the ‘infodemic’ culture where people would keep tabs on the progression of the disease in their community or globally [27, 48]. ‘Infodemic’, characterized by an excess of (mis) information on social media, has been linked with poor mental well-being and a significant increase in fear-induced behaviors along with anxiety, symptoms of depression, and post-traumatic stress disorder [25, 79]. Features of self-representation have also been identified as a significant cue leading to worsening of depressive symptoms through social media platforms, especially in participants who were more active through ‘photo-sharing’ activities on a daily basis [65]. Interestingly, the present findings revealed that 95.8% of participants used their mobile phones to access the Facebook platform, device which was found to be associated with the threefold heightened risk of addiction among young adults in Bangladesh [34]. Lastly, this increased Facebook dependency during the pandemic can be further mapped onto the Social Media Self-Control Failure framework, whereby a lack of focus on work can be translated to procrastination, frustration, and ultimately internal aggression on virtual platforms [32].

### Facebook and mental well-being post the COVID-19 mediated confinement

A shift was observed with a significant confinement-mediated effect on mood and feelings as demonstrated by a significant agreement (> 80%) to items such as ‘miserable and unhappy’ or ‘feeling lonely’ during confinement, contrasting with a general disagreement with similar feelings prior to and post confinement (< 5%); further interpreted as an increase in depressive symptoms initiated due to the confinement measures which were normalized after the lifting of those restrictions. Boursier et al. [9, 10] attributed this discrepancy between pre, during, and post confinement phases to feelings of loneliness, heightened levels of anxiety levels and excessive use of social media which further highlights the importance of the current study. Interestingly, this drastic ‘recovery’ post the lifting of sanitary measures can be superimposed on Skinner [74]’s theory of reinforcement [19, 75], whereby the removal of particular stimuli, which in this case, would be the laxation of sanitary restrictions, led to positive outcomes represented by decreased isolation and anxiety. Additionally, in line with the results, Meda et al. [47] report that lockdown restrictions may have exacerbated mental health problems, and upon lifting off those restrictions, any alterations in obsessive–compulsive and anxiety symptoms quickly diminished. Cao et al. [13] also pressed on the importance of adequate social support and familial relationships post the confinement on mental well-being. In this particular study, the re-appraisal of events as posited by Marzouki et al. [45] would be indicative of the re-evaluation of the sanitary conditions based on the most recent information and increased knowledge pertaining to the COVID-19 pandemic. Coupled with the ability to access social support through alleviation of the movement control, this would endorse the adjustment observed and encourage the normalization of mental health attributes.

The moderate levels of loneliness observed during the imposed social isolation directly predicted reduced engagement in healthy coping behaviors while the increased use of social media, especially Facebook, during confinement led to consequential changes to their emotional states which were confirmed by Moore and March [49]. Unfortunately, since confinement was synonymous with loneliness for most individuals as postulated by Swami et al. [80] and Ammar et al. [1], people experiencing loneliness were more likely to report higher levels of depression, poorer mental health in general; justifying the outcomes of this research. The rapid stabilization in mental well-being could also be addressed by the extensive usage of Facebook and other social media platforms as a means to maintain social interactions ‘live’ even under conditions of restrictive movements [52]. Hence, Facebook dependence could confer a certain degree of protective effect on its users by preserving the social capital through virtualization.

## Conclusion

This study probed into Facebook dependence and mental well-being before, during, and after the COVID-19 mediated confinement among Mauritian youth, aged 18 to 32 years old. An upsurge in Facebook usage was linked to a deterioration in mental well-being coupled with self-reported Facebook addiction, anxiety, sadness, loneliness, and depressive symptoms soaring considerably during the lockdown. The removal of several sanitary measures meant a slow adaptation into a ‘new normal’ phase which was synonymous with a remarkable recovery in mental well-being. The study provides significant pointers on how isolation reinforced the belief that heightened Facebook usage leads to detrimental psychological impacts. It is important to note that while the findings link heavy dependence to adverse mental well-being; it does not portray the role of the platform as a perpetrator of adverse psychological conditions, but rather demonstrate how individual usage patterns based on the type of content and overall time spent during socially restrictive conditions could precipitate changes in psychological states. Therefore, it is vital to raise awareness concerning the effects of the ‘infodemic’ culture as well as the detrimental impacts of Facebook misuse during conditions of social isolation and restrictive movements induced by a pandemic as a preparedness plan to curtail future burdens on mental health.

## Research gaps study and future directions

Given the ‘new normal’ circumstances, scales mapping pre-pandemic and post-pandemic sentiments are yet to be developed, hence, questions were contextually formulated for this study while pre-existing scales were adapted to the Mauritian landscape. Considering that the scales were previously used in Indian studies, the limitations were not significant as the Mauritian culture bears a consequential similarity with the Indian culture. Additionally, the impacts of the pandemic, without the influence of Facebook’s fluctuated usage, such as self-imposed isolation and compulsory loneliness might have acted as confounding effects on the mental well-being of participants. Moreover, how excessive use of Facebook psychologically impacts respondents has not been thoroughly explored in relation to their state of mind, despite its descriptive presentation. Since none of those respondents underwent a psychological evaluation prior to their participation in this study, no information was gathered regarding the potential pre-existing mental disorders and physical health conditions, which complements the idea of psychological resilience as a protective factor against Facebook addiction [31]. Hence, participants illustrating negative psychological states during the pandemic might have been due to pre-existing mental health conditions. Accordingly, future studies could tackle that discrepancy through control groups and using significant pointers such as social support and/or pre-existing mental conditions to investigate the impact of social media on the mental wellbeing of participants pre, during, and post confinement. Moreover, given the rise of prosocial behavior through online platforms [15, 18, 50]; the shift towards virtual frameworks supporting consumer behavior [88], and e-learning platforms due to the pandemic [43, 82], the importance of social media has been redefined. The impact of such measures gives way to further research in relation to social media and mental wellbeing post the pandemic.

## Supplementary Information

Below is the link to the electronic supplementary material.

Supplementary file1 (docx 17 Kb)
