# Supplementary material for: Living through the COVID-19 pandemic in Mauritius: mental well-being and dependence on Facebook
Source: Discov Psychol. 2022 Jul 1;2(1):32. doi: 10.1007/s44202-022-00044-4 (PMC10115481; doi:10.1007/s44202-022-00044-4)
Supplement: Supplementary file 1 — Supplementary file1 (docx 17 Kb) [file 44202_2022_44_MOESM1_ESM.docx]

**Supplementary Data S1:**

**Table S1. Assessing mental state of participants through the Short Mood and Feeling Questionnaire**

|  | **Pre-Confinement** | | | **During Confinement** | | **Post Confinement** | |
| --- | --- | --- | --- | --- | --- | --- | --- |
| **Description** |  | **Freq** | **%** | **Freq** | **%** | **Freq** | **%** |
| Miserable and unhappy? | Yes | 11 | 2.8 | 339 | 84.8 | 15 | 3.8 |
|  | No | 367 | 91.8 | 18 | 4.5 | 341 | 85.3 |
| Did not enjoy anything at all? | Yes | 8 | 2.0 | 338 | 84.5 | 15 | 3.8 |
|  | No | 370 | 92.5 | 18 | 4.5 | 341 | 85.3 |
| .I felt so tired.(I did nothing) | Yes | 15 | 3.8 | 350 | 87.5 | 14 | 3.5 |
|  | No | 363 | 90.8 | 9 | 2.3 | 343 | 85.8 |
| Very Restless? | Yes | 11 | 2.8 | 345 | 86.3 | 12 | 3.0 |
|  | No | 367 | 91.8 | 11 | 2.8 | 344 | 86.0 |
| Felt not good at all? | Yes | 11 | 2.8 | 338 | 84.5 | 14 | 3.5 |
|  | No | 367 | 91.8 | 19 | 4.8 | 339 | 84.8 |
| I cried a lot. | Yes | 9 | 2.3 | 302 | 75.5 | 8 | 2.0 |
|  | No | 369 | 92.3 | 57 | 14.3 | 350 | 87.5 |
| Found it hard to think properly/concentrate? | Yes | 11 | 2.8 | 331 | 82.8 | 15 | 3.8 |
|  | No | 367 | 91.8 | 27 | 6.8 | 340 | 85.0 |
| I hate myself. | Yes | 8 | 2.0 | 301 | 75.3 | 10 | 2.5 |
|  | No | 370 | 92.5 | 58 | 14.5 | 347 | 86.8 |
| I felt lonely | Yes | 9 | 2.3 | 331 | 82.8 | 9 | 2.3 |
|  | No | 369 | 92.3 | 29 | 7.3 | 348 | 87.0 |
| I thought nobody really loved me. | Yes | 14 | 3.5 | 327 | 81.8 | 13 | 3.3 |
|  | No | 364 | 91.0 | 31 | 7.8 | 340 | 85.0 |
| I did everything wrong | Yes | 9 | 2.3 | 325 | 81.3 | 11 | 2.8 |
|  | No | 369 | 92.3 | 34 | 8.5 | 342 | 85.5 |

*Data presented in Frequency (n = ) and percentage (%) based on total number of respondents for the individual items.
